# An Approach of Query Audience’s Attention in Virtual Speech

**DOI:** 10.3390/s24165363

**Published:** 2024-08-20

**Authors:** Hongbo Kang, Rui Yang, Ruoyang Song, Chunjie Yang, Wenqing Wang

**Affiliations:** School of Automation, Xi’an University of Posts and Telecommunications, Xi’an 710061, China; khb2000@xupt.edu.cn (H.K.); yang.xupt.edu.cn@stu.xupt.edu.cn (R.Y.); sry@stu.xupt.edu.cn (R.S.); ycj@xupt.edu.cn (C.Y.)

**Keywords:** attention evaluation, gaze tracking, EEG, virtual speech, eye contact

## Abstract

Virtual speeches are a very popular way for remote multi-user communication, but it has the disadvantage of the lack of eye contact. This paper proposes the evaluation of an online audience attention based on gaze tracking. Our research only uses webcams to capture the audience’s head posture, gaze time, and other features, providing a low-cost method for attention monitoring with reference values across multiple domains. Meantime, we also propose a set of indexes which can be used to evaluate the audience’s degree of attention, making up for the fact that the speaker cannot gauge the audience’s concentration through eye contact during online speeches. We selected 96 students for a 20 min group simulation session and used Spearman’s correlation coefficient to analyze the correlation between our evaluation indicators and concentration. The result showed that each evaluation index has a significant correlation with the degree of attention (*p* = 0.01), and all the students in the focused group met the thresholds set by each of our evaluation indicators, while the students in the non-focused group failed to reach the standard. During the simulation, eye movement data and EEG signals were measured synchronously for the second group of students. The EEG results of the students were consistent with the systematic evaluation. The performance of the measured EEG signals confirmed the accuracy of the systematic evaluation.

## 1. Introduction

Attention refers to an individual’s conscious and selective focus on specific information or stimuli. In psychology and cognitive science, attention is a complex mental process involving selective perception, information filtering, and the allocation of attentional resources. It encompasses the directed focus on particular information within internal thoughts and the external environment, as well as the ability to switch attention between different stimuli. The rational allocation of attention is crucial for understanding and processing information, particularly in activities such as communication and learning. Eye contact is a vital clue of attention during offline/in-person communication. Online teaching, virtual meeting, and other virtual speeches are very popular ways of communication nowadays, but this way has a huge defect that lacking of eye contact between speakers and audiences. It is revealed by psychologists that the communication effect in daily life depend on 7% text, 38% sound, and 55% body language, furthermore sight language is an important part of body language. Eye contact plays an important role for both speakers and audiences. For a speaker, eye contact can convey goodwill and confidence, and initially establish a connection with audiences [1]; meanwhile, good eye contact can also mobilize the audience’s emotions and make speech more attractive and persuasive [1,2,3]. For audiences, eye contact can allow them to make a preliminary judgment of speakers, and it can also help them maintain their attention and increase their enthusiasm for the content of the speech [4]. At present, existing online conference platforms such as Zoom and Tencent Conference do not provide this technology. Speakers can only gauge the state of their audience’s attention through questions, tests, etc., which cannot achieve the same effect as offline conferences. Therefore, we need a way to make remote “eye contact” between speakers and audiences to obtain the audience’s degree of attention.

Common ways to achieve related functions include eye tracking data indicators and EEG signals. EEG signals have been proven to be one of the most efficient and accurate physiological indicators for evaluating attention levels [5], and can also be used to independently express their attention level. Alirezaei [6], when extracting features from raw EEG signals, found that the energy signature of the beta band can be used as an important reference information for evaluating the state of attention. Janelle analyzed the spectral characteristics of EEG signals and determined the level of attention in the brain by observing the magnitude of the ratio of theta and beta band power [7]. In recent years, with the continuous development of machine learning methods, more machine learning-based attention level evaluation methods have gradually emerged. Toa [8] used an EEG signal acquisition device to collect data on subjects’ attentional concentration and distraction behaviors during a trial, and analyzed the collected data to classify subjects’ attention levels through a convolutional attentional memory neural network (CAMNN) model; the results of the study showed that the model’s recognition accuracy was better than that of other existing neural network models. Wang et al. [9] used the power spectral density obtained via STFT as a feature and input it into a CNN-based personalized classifier, which achieved good performance in detecting subjects’ attention. In an online learning environment, Al-Nafian [10] evaluated students’ attention levels through EEG signals and effectively detected changes in their attention. Atilla [11] achieved the evaluation of the driver’s attention state by detecting their EEG signals, avoiding their driving. From the above research results, we can conclude that EEG will be a very effective way to evaluate the attention of audiences sitting in front of a computer. But during the measurement process, using EEG requires that one wears a wearable device. Thus, it is not suitable for use in daily life scenes. Eye tracking technology can record the trajectory of the audience’s eye gaze movement in real time, and analyze the gaze data without any contact with the audience. Molina [12] used the Tobii X60 gaze-tracking device to record the gaze points of students when viewing textbooks, and the result showed that students’ learning efficiency increased by about 40% when texts and related pictures were closer. Krstic [13] used the SMI RED-m eye tracker to collect the gaze points of 92 students, and found that students with a strong reading ability moved their eyes more smoothly. Wermeskerken [14] used eye-tracking technology to calibrate the audience’s gaze, and found that whether the speaker’s face appeared on the screen had no effect on speeches. Zhou [15] used the Tobii Pro X2-30 eye tracker to collect the gaze points of students when browsing the website, and found that there were differences in the ability to search for information between students with excellent grades and poor grades. Jian [16] used an eye tracker to record the gaze points of students while reading, and the result showed that students with good grades pay more attention to images and charts. Karch [17] collected the gaze points and pupil sizes of 22 listeners through the Tobbi X2-60 eye tracker, and found that, when answering more complex questions, the pupil sizes of the listeners would change. Fichtel [18] used the SMI REDn eye tracker to record the gaze area of the expert doctor during operation and used it to guide the general doctor’s surgery to reduce the risk of operation. These eye-tracking-related applications have achieved the estimation of the gaze points or gaze directions. Cibulska [19] proposed a virtual reality system suitable for online learning, which features multiple means of interconnectivity, student learning progress analysis, and communication among multiple users. These functionalities enhance interactions between students and the virtual environment. Chen [20] developed a virtual reality cultural heritage education game where users can experience the journey of the “Gotheborg” ship sailing to Guangzhou. This study integrates virtual reality technology, game design, and education to facilitate knowledge acquisition within the game. Misbhauddin [21] developed a VR framework based on the Unity 3D engine and handheld Bluetooth devices, enabling students to interact with virtual classrooms through relevant tools.

However, the above-mentioned studies did not achieve the analysis of user attention through eye-tracking data and relied on expensive equipment or complex accessories. Some of them are not suitable for daily life, and even harm users when used for long periods of time. To more widely meet the requirements of virtual speech, a good evaluating system should be low-cost, should run on an ordinary PC or mobile platform, and be comfortable when used for long periods of time and without external auxiliary equipment. Nandini [22] utilized eye-tracking technology on e-commerce portals to understand online consumer behavior. By tracking users’ gaze positions on the screen, they gained insights into their focal points and browsing behavior on webpages. This holds significant value for optimizing website design, enhancing user experience, and increasing sales conversion rates. In the study by Ashby [23], eye-tracking methods were employed to explore the relationship between consumers’ attention allocation to evaluations and their individual estimations of product value, aiming to predict consumers’ willingness to pay and willingness to accept. Conley [24] utilized eye tracking technology to assess the usability of Blackboard, an online learning management system. Through the analysis of eye-tracking data, they revealed students’ focal points, browsing patterns, and potential cognitive loads while using Blackboard, understanding the distribution of the attention and interaction behavior of users when browsing and utilizing the system. Through this approach, they identified which aspects of the system positively or negatively impacted students’ learning experiences, thus improving the system design and enhancing its usability. The research solely relied on webcam-based gaze tracking without incorporating the functionality of analyzing user attention through eye-tracking data. As a result, it may not be suitable for virtual speech. This paper proposes an evaluating attention system based on gaze tracking to solve the above problems. The system uses convolutional neural networks to predict gaze features instead of using expensive eye-tracking equipment and can run on home computers. In addition, we defined an index system to evaluate the attention of audiences by combining behaviors such as head pose and eye movement. Since the real state of the audience is unknown, the evaluation results of the attention evaluation system may not be reliable. Therefore, we verified the evaluation results with the objective and accurate characteristics of EEG. In this way, the system makes up for the speaker’s inability to gauge the audience’s attention through eye contact, and achieves remote “eye contact”.

## 2. Related Work

### 2.1. Research on the Role of Eye Contact in Speech

Scientific research has found that when two people look at each other for a long time, the activity of mirror neurons will occur, and one party will be affected by the emotions of the other party; therefore, eye contact has an important impact on the effect of speech [25]. When an audience shares the same opinions as the speaker, they will spend more time looking into the speaker’s eyes and are more likely to accept the speaker’s opinions, and if the speaker looks directly into the eyes of their audience instead of avoiding their eyes, this sense of identity can be deepened [26]. Excellent teachers have 15% more eye contact than novice teachers. Hains-Wesson found in a survey that 93% of students thought eye contact had a very important impact on their learning; 91% agreed that eye contact could help them maintain their focus; and 74% believed that eye contact could increase their enthusiasm for knowledge. Masanori [27] used factor analysis to explore the role of eye contact in online teaching, and found that, with eye contact, students’ ability to understand knowledge in online teaching is basically the same as in offline teaching; but, without eye contact, students need to make a greater effort to understand the content of the speech. Carolyn [28] also studied the role of eye contact in online teaching, and found that students’ attention and curiosity levels were related to whether they made positive eye contact with teachers. Students who make eye contact with teachers make higher learning efforts, encounter fewer difficulties, and achieve better learning results; meanwhile, they found that students with good grades are better at receiving the information conveyed by the eyes. Fiorella [29] and Beege [30] also conducted related research, and in their experiments, groups with eye contact achieved better learning outcomes. Ouwehand [31] tracked the eyes of an undergraduate group in class, and they found that when teachers are visible in a video, the college students will focus most of their attention on the teacher’s face. Therefore, teachers can use eyes to guide students to pay attention to the knowledge points in learning materials. Walters [32] believed that speakers can check that everyone is concentrating. Snyde [33] thought speakers can hold the attention of students not being addressed and encourage them to listen to knowledge seriously. Zeki [34] also believed that eye contact can set the tone of a lesson, and teachers can use eye contact to know that students are ready for class. These studies have shown that, in online speeches, eye contact between speakers and audiences has an important impact on the effect of speech, but they did not collect and analyze the eye movement data in the classroom. Wagner [35] used the eye-tracking headset to detect the impact of the teacher’s gaze proxy on the learning effect of the students, but he did not analyze the relationship between eye movement data and attention, and relied on additional equipment. Ding [36] proposed GazeReader, an unknown word detection method only using a webcam, and provided a good idea for positioning the sight, but he needed to train a more complex language model to capture context information.

### 2.2. Selection of Eye Feature Acquisition Method

There are four common methods to obtain human eye features, namely gaze tracking based on geometry, gaze tracking based on a three-dimensional eye model, gaze tracking based on eye appearance features, and gaze tracking based on deep learning

Geometry-based gaze tracking uses the position changes of eye feature points (such as infrared spots, eye geometry, etc.) within the eye range to estimate gaze. It is usually difficult for such methods to deal with head movement, which greatly limits their application areas. Gaze tracking based on the 3D eye model obtains the gaze vector through the 3D modeling of the eye structure, and then obtains the fixation point by calculating the intersection point of the gaze vector and screen. This method greatly reduces the impact of head motion on the prediction results, but require higher hardware precision. Gaze tracking based on eye appearance features processes the collected images to obtain the characteristic parameters of eyes and then realizes the gaze estimation. Such methods do not require additional hardware facilities, but are less adaptable to head movements or changes in the external environment, and it is difficult to cope with the various situations that may arise in online education. The comparison between existing gaze-tracking algorithms is shown in Table 1.

The eye-tracking method based on deep learning collects eye images in multiple directions, with multiple angles and different lighting environments, and uses the convolutional neural network to establish the relationship between eye images and screen fixation points. Compared with the shortcomings of traditional gaze tracking, which is affected by factors such as head pose and lighting conditions, gaze tracking based on deep learning is more suitable for online speech. Not only does this method not require calibration work, but the equipment is simple, requiring only an ordinary home network camera. And when the training data are sufficient, the line of sight can be accurately detected. Therefore, we finally select a gaze-tracking method based on deep learning to achieve the acquisition of eye features. Currently, widely used databases include MPIIGaze [37], GazeCapture [38], and so on.

## 3. System Structure

The main body of the system is divided into two parts. The first part uses the captured image frames to extract the features of the face and eye. The second part uses the convolutional neural network model to estimate the gaze point and gaze direction combined with the head pose. This system does not require additional hardware equipment, and it can greatly reduce the impact of head rotation, so it is suitable for any online speech platform.

### 3.1. Acquisition of Facial Features and Eye Features

In order to adapt to the capture of human faces in different environments, the system combines skin color detection and face detection. Skin color detection uses the Otsu adaptive threshold algorithm based on the YCrCb space to remove pixels other than skin color in images, and then uses the Haar-Adaboost classifier [39] to detect faces on it, which greatly reduces the external noiseThen, the key point detection is performed on the detected face frame to locate the accurate shape of the face. This system uses the GBDT algorithm [40] for face alignment, and this algorithm can realize the positioning of 68 key points on the face within milliseconds, and its accuracy is high, no less than the traditional face alignment algorithm. The full name of GBDT is the gradient boosting decision tree, which is a traditional machine learning algorithm. Its basic idea is to add the results of all weak classifiers as the predicted value, and then the next weak classifier is used to fit the residual of the predicted value (this residual is the error between the predicted value and the real value), and the final result is obtained by adding the results of the classifiers in each layer according to weights. The final strong classifier model is shown in (1).

(1) $$f\left(x\right)=\sum_{m-1}^{M}\beta_{m}b\left(x,\Upsilon_{m}\right)$$

where *b*(*x*;$\Upsilon_{m}$) is the weak classification model, $\beta_{m}$ is the weight coefficient of the weak classification model, and $\Upsilon_{m}$ is the parameter of the weak classification model.

**Figure 1:**
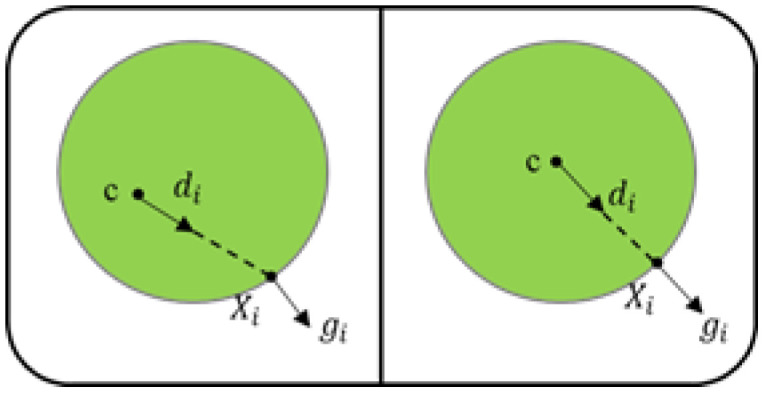
Locating the pupil center. $g_{i}$ is a unit vector pointing to the edge point $x_{i}$, $d_{i}$ is the unit vector pointing from *c* to $x_{i}$, and the pupil center c∗ is the point with the largest sum of dot products of $d_{i}$ and $g_{i}$ among all *c*.

The last is the positioning of the pupil center. The pupil center is not only an input feature required by the gaze estimation model, but also plays an important role in evaluating attention. We chose the pupil center location algorithm based on the Hough transform [41,42]. This method takes advantage of the convex feature which the pupil center has and considers that the gradient vector of the pupil edge points to the opposite direction of the line connecting point and the pupil center, as shown in Figure 1.

**Figure 2:**
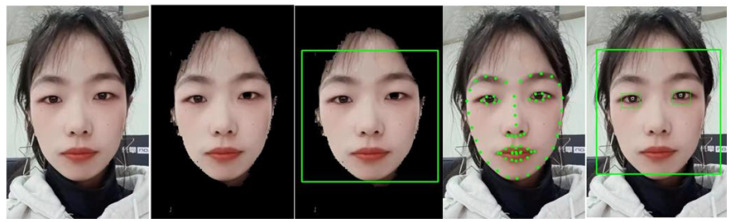
Extraction of facial features and eye features. The first is the original image, the second is the image which remove pixels other than skin color, the third is the result of face detection, the fourth is the detection of face alignment, and the fifth is the result of pupil center positioning.

We traverse the black pixels in the eye image, make a unit vector $g_{i}$ pointing to the edge point $x_{i}$, and calculate its dot product with the gradient vector $d_{i}$ of point $x_{i}$. The center of pupil $c^{*}$ is the largest sum of dot products with all edge points, and the calculation formula of $c^{*}$ is shown in (2). This method locates pupils within 3 pixels of error. The example of obtaining facial features and eye features is shown in Figure 2.

(2) $$c^{*}=argmax\left\{\frac{1}{N}\sum_{i=1}^{N}{\left(d_{i}^{T}g_{i}\right)}^{2}\right\},d_{i}=\frac{x_{i}-c}{∥x_{i}{-c∥}_{2}},\forall_{i}:{∥g_{i}∥}_{2}$$

### 3.2. Gaze Tracking

**Figure 3:**
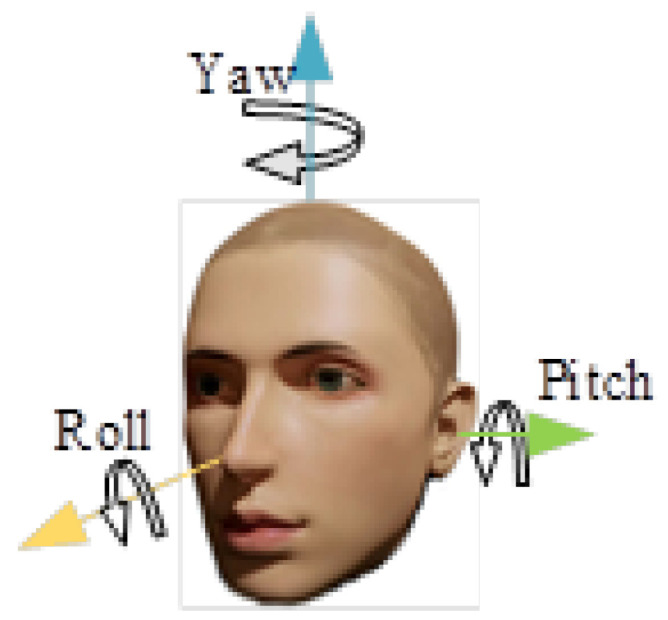
Head pose display. Pitch, yaw, and roll are the Euler angles that rotate around the X axis, Y axis, and Z axis, and the head post can be marked according to these three angles.

In 3D space, the head pose can be represented by three Euler angles of pitch, yaw, and roll, as shown in Figure 3. The head pose is used for gaze tracking. We used the general head 3D model [37], and obtained the conversion formula from the screen coordinate system to the world coordinate system using the EPnP algorithm [43,44]. The EPnP algorithm is used to compute the camera pose, and it estimates the position and orientation of the camera relative to an object using known 3D points on the object and their corresponding 2D projection points in the image, the camera pose is represented by a rotation matrix R and a translation matrix T. The relationship between the 3D points on the object and their corresponding 2D points in the image can be described by Equation (3); by substituting specific points, we can obtain the rotation matrix R and translation matrix T.

(3) $$S\left[\begin{array}{c} x\\ y\\ 1 \end{array}\right]=\left[\begin{array}{c} f_{x}\\ 0\\ 0 \end{array}\right]\left[\begin{array}{ccc} f_{x} & 0 & c_{x}\\ 0 & f_{y} & c_{y}\\ 0 & 0 & 1 \end{array}\right]\left[R|T\right]\left[\begin{array}{c} U\\ V\\ W\\ 1 \end{array}\right]$$

where *R* is the rotation matrix, *T* is the translation matrix, and the intermediate matrix is the internal reference matrix of the camera. We can use the rotation matrix to obtain the three Euler angles of the head pose.

**Figure 4:**
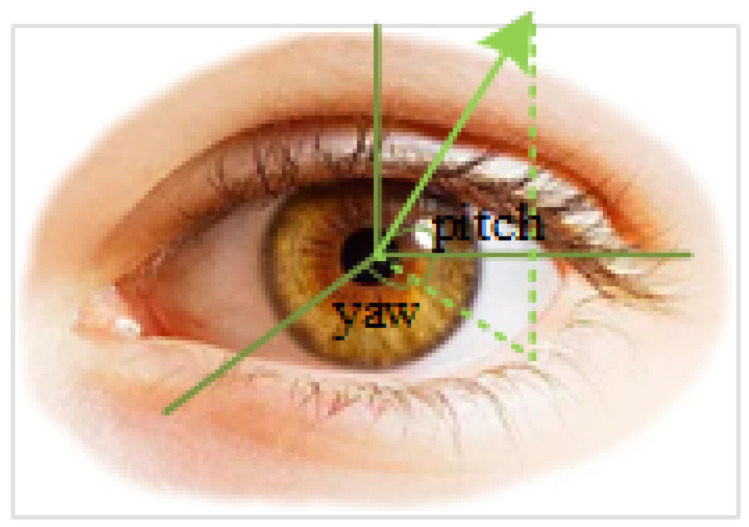
Gaze direction. Pitch and yaw are the Euler angles that rotate around the X axis and Y axis, and the gaze direction can be marked according to these two angles.

**Figure 5:**
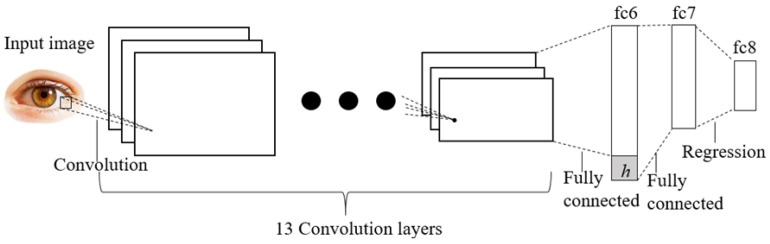
Architecture of GazeNet. The input of the network is a monocular image, and after being operated and flattened by thirteen layers of convolution, it concatenates with the head pose in the first fully connected layer, and the output is the gaze direction vector (pitch and yaw) of the eyes.

The next step is to estimate the gaze direction, which is to judge the gaze direction from the captured video frames. The direction can be described by pitch and yaw, as shown in Figure 4. We use the GazeNet [37] to estimate it, as shown in Figure 5 along with the CNN network architecture. The network extracts deep appearance features based on the VGG-16 deep convolutional neural network [45]. This is a way that estimates the gaze of one eye, that is, the gaze directions of the two eyes are estimated separately.

We use the sight with both the pitch and yaw in the range [−30°, 30°] as a positive, and sight outside this range as a negative. We set that, when the error between the real sight and the predicted sight exceeds 5.9° [37], the predicted result is wrong. Then, we use the MPIIGaze [37] dataset to test the model, and obtain its confusion matrix as shown in Table 2. Obviously, the model can make a high-accuracy prediction of the audience’s gaze direction.

**Figure 6:**
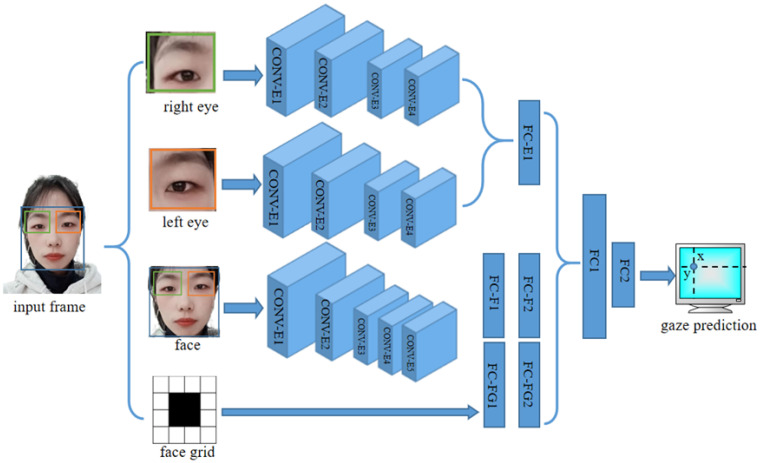
Architecture of the GazeNet. The input of the network is a monocular image, and after being operated and flattened by thirteen layers of convolution, it concatenates with the head pose in the first fully connected layer, and the output is the gaze direction vector (pitch and yaw) of the eyes.

The last step is gaze point estimation. The gaze point estimation serves to estimate the point where the gaze is focused on a two-dimensional plane. This plane can be a mobile phone, pad, computer, etc. We implemented this task using the iTracker [38] network. iTracker is a convolutional neural network for eye-tracking. The overall architecture of the network is shown in Figure 6. Although the face image already contains the eyes, the eye image is still used as a separate input to obtain a more accurate image.

We set the gaze point in a 8 cm × 8 cm square area in the center of the screen as positive, the points in other ranges as negative, and the error threshold as 1.69 cm [39]; then, we use the GazeCapture [38] dataset to test the model, and obtained its confusion matrix, as shown in Table 3.

## 4. Evaluating Concentration

### 4.1. Index System

In order to evaluate the attention of audiences in an online speech, a set of indexes should be set up first. According to Zhang [46] and Klein [47], when an audience paid close attention to a speaker, their gaze showed three main movements: fixation, saccades, and smooth pursuit. Fixation refers to a gaze focusing on a certain observation point and lasting at least 100~600 ms. Most information is generally obtained in this movement. Saccades refers to the rapid transfer of one’s gaze from one fixation point to another. The angle between two points is generally 10~40°, and this lasts 30~120 ms—it is usually difficult to obtain any information in this movement. Smooth pursuit refers to movement process wherein one’s gaze follows a target with an angle change from 1~30° per second, and it is generally not executed when there is no target. So, to summarize the above in relation to our experimental observation, we propose a set of indexes for the evaluation system as follows:

- The proportion of video frames with one face (POOF) is more than 95%.
- The proportion of video frames with a high concentration head pose (POHCHP) is more than 50%.
- The proportion of video frames with a medium concentration head pose (POMCHP) is more than 90%.
- The proportion of the effective fixation time to the total fixation time (POEFT) is more than 90%.
- The proportion of serious and effective fixation time to total fixation time (POSEFT) is more than 50%.
- Gazing at pictures and charts (GPC) accounts for more than 30% of the total gazing time.
- Gazing at teachers’ facial images (GTFI) accounts for more than 20% of the total gazing time.
- The variance in the number of saccades (NOS) per 100 frames is less than 10.
- There are more than five instances of reading smooth pursuit (NORSPs) every ten minutes.

Of course, the degree of attention that an audience pays to a speaker involves much more than the above, which means that certain factors may not be obtained or cannot be determined by conventional equipment. This work is based on these essential factors which may serve practical functions in daily life.

### 4.2. Process of Evaluation

**Figure 7:**
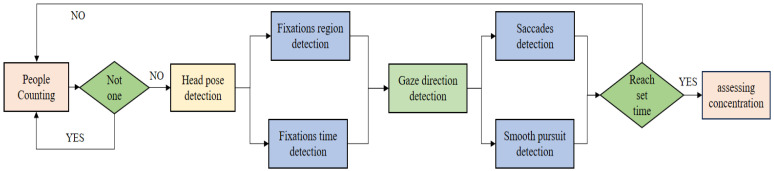
The process of evaluating attention. Firstly, identify the number of people in the image, if the number is not one, then re-identify. Secondly, the eye movement data, including the user’s gaze range, gaze time, gaze direction, number of saccades, and reading smooth pursuit times are counted for a period of time, and the user’s attention is evaluated through these data.

The process of evaluating attention is shown in Figure 7. Since the running speed of systems will be affected by the performance of computers, if a fixed time period is selected for analysis, it may lead to uncertainty. Therefore, we choose to analyze every 100 frames and evaluate every 10 min.

First, check the number of faces on the screen. Since most of the online teaching attendees are single-person and single-device, and some teaching resources are paid resources, it is forbidden for multiple people to watch on the same device. Therefore, when it is detected that the number of faces in the video frame is not 1, check the number of faces again until the number is 1.

Next, the detection of the head pose is carried out. We believe that when a person is in a state of concentration, the head will keep facing the direction of the screen and rarely turn significantly. Therefore, when it is detected that the absolute values of yaw and pitch are both in the range of [0°, 10°], it is considered that the audience is in high concentration, and when one of the two value is in the range of (10°, 30°] and the other is less than 30°, it belongs to medium concentration, while when one of these is in the range of (30°, 90°], it belongs to low concentration [48].

Then, the fixation detection is performed, and we judge a behavior in which the gaze stays in a small area for more than 500 ms as a fixation.

**Figure 8:**
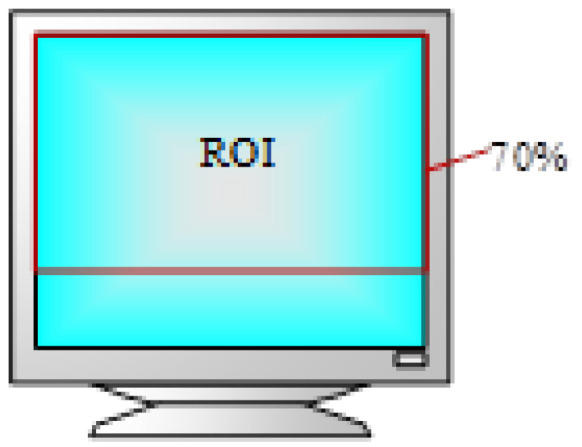
Area range of ROI. ROI is defined as the top 70% of the screen.

The experimental results of Owens [49] show that the attention of lecturers is mainly concentrated on the upper part of their screen, and the number of times they look down is less, so we use the top 70% of the screen as the ROI area, as shown in Figure 8. When the fixation point of the lecturer falls inside the ROI area, the fixation is considered to be effective fixation. When the fixation point falls outside the ROI area, it is considered to be invalid.

We recorded the duration of each fixation. According to the mind–eye hypothesis [50], long-term fixation means that attention is devoted to difficult situations. Therefore, when the fixation point is located inside the ROI area and the fixation duration is longer than 3 s, it is considered as a serious and effective fixation. We determine the fixations by comparing the gaze direction and fixation points between the adjacent frames in the program. If the fixation positions in two consecutive frames are the same, we generate a temporary data record for the duration of this fixation at that point. Then, based on the duration and position, we determine whether to include it as an effective fixation time or as a serious and effective fixation time. We do not need to record the fixation time for each point, and only the total duration of the effective fixation time, total duration of serious and effective fixation time, and total fixation time are required. Philip [51] proposed that, when faced with pictures and diagrams, the eyes will have a natural tendency to wander over these things. When the ppt appears on a screen, people’s gaze will unconsciously move towards them. Therefore, we believe that an attentive listener will spend a certain percentage of time acquiring information from pictures and diagrams. Wechsler [52] believes that the degree of attention can be represented by the time that gaze is focused on the speaker’s face, so we also take the length of it as part of our evaluation index.

Finally, gaze movement detection is performed. The gaze movement is divided into saccades and smooth pursuit. We compare the gaze angle between two consecutive video frames. If it is greater than or equal to 10°, it is judged as saccades. If it is less than 10°, it is smooth pursuit. We made statistics from the eye movement data of 10 students in every 100 frames when they were studying seriously and performing recreational activities, and found that the mean value was not much different. However, the variance in eye movement data was only 6.55 when studying seriously, and 14.10 when performing recreational activities. Therefore, we believe that a student who is in a state of concentration will have more stable saccades data per unit time. We also distinguish the smooth pursuit according to the Western reading habit from left to right, and regard the smooth pursuit from left to right with a small up and down amplitude as reading smooth pursuit.

### 4.3. EEG Processing

**Figure 9:**
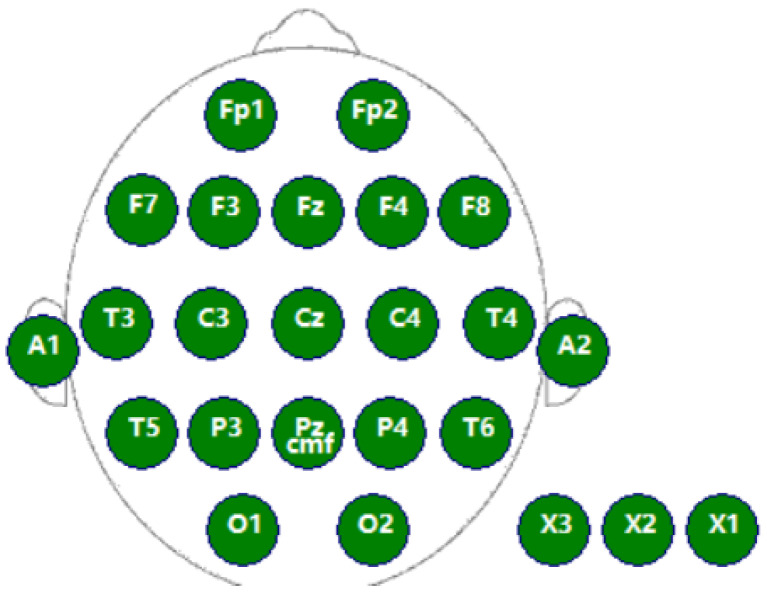
Distribution of EEG sensors.

In order to validate the results, we employed an electroencephalograph to measure EEG signals and objectively verify participants’ evaluation outcomes. Attention evaluation using EEG signals can be performed using machine learning-based attention evaluation methods, as well as traditional methods of observing power changes in the specific frequency bands of the EEG. Van Son [53] analyzed the EEG data of the respective channels of the prefrontal, central, and parietal frontal in the frontal regions by experimental control and concluded that the power changes in the prefrontal (F3, Fz, F4) were more closely related to the level of attention. Clarke [54] found that the beta band power of EEG signals increases and that the theta band power energy decreases when people are in a state of focused attention. Mind wandering refers to the emergence of task-unrelated influences that draw attention away from the task at hand [55]. Mind wandering is directly related to reduced attention and focus [56,57]. Braboszcz and Delorme demonstrated that EEG power in the beta band is lower and *θ* band power is higher in the mind-wandering state [58]. In the present study, the electrode channel data were collected for the frontal (F3, Fz, F4) positions of the subjects in accordance with the previous experience. Based on the change in the mean value of the frontal electrode (F3, Fz, and F4) power in the first half of the time and in the second half of the time, it was determined whether the attention was altered in the two periods of time. The electroencephalograph was a wireless DSI-24 dry electrode EEG system developed by Wearable Sensing, USA, with a sampling frequency of 300 Hz. The system can include two parts, namely hardware and software. The data acquisition software, DSI-Streamer–v.1.08.44, can record raw data and output csv and edf file formats. The hardware component is the EEG sensors, and there are 20 sensors that have been installed in a lightweight, user-adjustable headset that positions the sensors at the Fp1, Fp2, F7, F3, FZ, F4, F8, T3, C3, CZ, C4, T4, T5, P3, P4, T6, O1, and O2 positions of the International 10/20 standard system, as shown in Figure 9.

**Figure 10:**
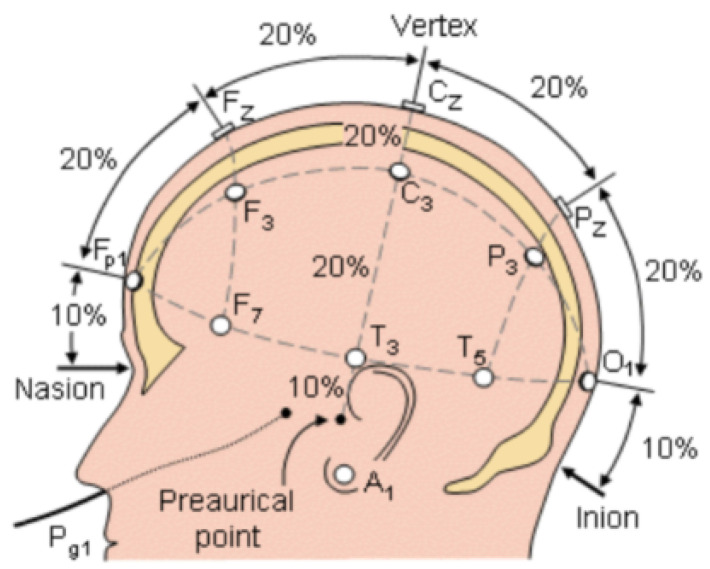
Distribution of EEG sensors.

As shown in Figure 10, the 10–20 system divides the head into regions, where 10% and 20% represent the relative proportions of the distance from the head suture to the tip of the nose and the posterior cranial eminence to the virtual vertical line.

**Figure 11:**
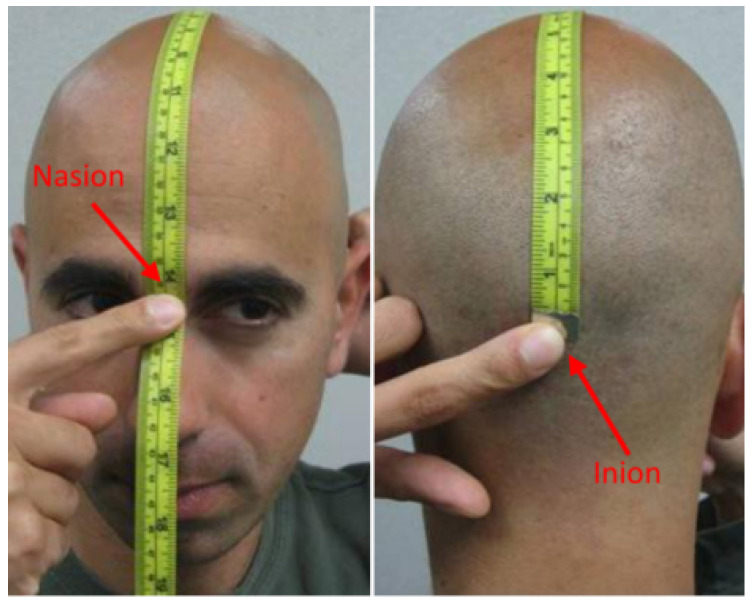
How to locate and measure the nasion, inion, and CZ positions on the head.

The CZ and FZ sensors were placed on the head. Subjects wore the headphones in a manner ensuring that the FZ sensor was aligned with the forehead arrow and the C3 and C4 sensors were aligned with the ear holes. Tension also needed to be adjusted by pushing down and rotating the sensors through the hair to keep the headphones close to the scalp using the tool provided. The crown piece is correctly oriented by accurately positioning the CZ sensor. CZ is situated as shown in Figure 11, exactly halfway between the nasion and inion points. Calculate the distance between the nasion and the front edge of the FZ sensor using the formula 0.5 × (nasion–inion) = 9.3 cm.

The EEG signals were processed offline using the eeglab tool in Matlab. Since the spectral power of the EEG decreases rapidly with increasing frequency, the ultra-low-frequency component of the EEG (<1 Hz) often dominates the entire spectrum, so a high-pass filter was applied at 0.1 Hz, and a 50 Hz trapping filter was applied to remove the interference from the industrial frequency. These data were automatically corrected for ocular artifacts at 4 s intervals. Residuals containing muscle movements, amplitudes above 200 µV, or other artifacts were removed.

The collected EEG signals in this study are voltage-based. To derive the power of a specific frequency band, the integration of the power spectral density within the required frequency range is necessary. Numerous methods exist for estimating power spectral density. The periodic graph method is the simplest and fastest approach; however, its estimated variance increases with sampling points. To overcome these limitations, we utilize the Welch method as a classic spectrum estimation technique that provides relatively accurate estimations of power spectral density. Hence, we adopt the Welch method for our power spectral estimation process. The calculation steps are outlined as follows:

(1) Divide the M samples of the signal into L data segments (overlapping), where each data segment contains N samples; then, M = LN, and D sampling points overlap between two adjacent data segments;
(2) Windows are added to the data segment, and the power spectral density of the data after Windows is calculated as P(f) by the period-graph method. The formula for calculating the power spectral density of section k is as follows:
  (4) $$P_{K}\left(f\right)=\frac{1}{NU}|\sum_{n=1}^{N}x\left(n\right)w\left(n\right)e^{-j2\pi fn}{|}^{2}$$
  In the above formula, $P_{XX}\left(f\right)=\frac{1}{N}\sum_{1}^{N}w^{2}\left(n\right)$ is the normalization factor and x(n) is the timing signal; w(n) is the window function; and *N* is the signal number.
(3) After obtaining the power spectral density of each data segment, the period graph of all windowed data segments is averaged, and the average value is used as the final power spectral density estimate
  (5) $$P_{xx}\left(f\right)=\frac{1}{L}\sum_{k=1}^{L}P_{k}\left(f\right)$$
  To visualize the variation of power over frequency, we logarithmically process the calculated power.
  (6) $$P_{welch}\left(f\right)=10*log10\left(P_{xx}\left(f\right)\right)$$
  At this point, we sum $P_{welch}\left(f\right)$ over a specific frequency range, and then take the average as the basis for our evaluation. All the above operations can be realized in Matlab.

## 5. Tests

### 5.1. Test Content

The test site was chosen to be in a well-lit conference room. Ninety-six current college students (75 males) participated in this study. Participants had to be between 18 and 24 years old, and they had an average age of 21.8 years. Subjects were free from anxiety as well as attention disorders, and were also required to wash their hair on the day of the experiment, and not to consume beverages or medications that affect their mental state for 24 h prior to the experiment. The equipment used was a LENOVO ideaPad 300-15 notebook computer, which uses the Windows10 HomeBasic 64bit operating system, has a screen size of 15.6 inches (1366 × 768 pixels), and a CPU of 2.30 GHz. The camera is 130 W pixels, which is not a high-precision one. The EEG equipment is a wireless DSI-24 dry electrode EEG system developed by Wearable Sensing, San Diego, CA, USA, with a sampling frequency of 300 Hz. A motorized brush was used to clean the sensor with isopropyl alcohol or 70% ethanol before turning on the unit and air-drying the sensor.

The participants in the experiment were 96 graduate students. We conducted two tests of system error test and a classroom simulation to test the performance of the system and our hypothesis. Since existing technology does not have the function of identifying who the speaker is and locating their facial image among all the portraits on screens, and cannot accurately identify the pictures and diagrams related to their speech, we only collected six indicators, namely POOF, POHCHP, POMCHP, POEFT, POSEFT, NOS, and NORSP. Also, due to the limited number of EEG devices, we only measured the EEG signals of 32 students, and only Bata (13–32 Hz) and theta (4–8Hz) band power values were extracted from their foreheads (FZ, F3, F4 electrodes).

### 5.2. Ethics Statement

All our research involving human participants complied with the ethical standards set by the Declaration of Helsinki and those set by various relevant institutions. All subjects and/or their legal guardian(s) provided informed consent for participation in the study and the publication of identifying images in an online open access publication. Our experiments were approved by the Institutional Review Board (IRB) of Xi’an University of Posts and Telecommunications.

### 5.3. System Accuracy Test

We first tested the system performance to obtain the accuracy of the system in different environments for the recognition of various features. The impact of the different lighting conditions tested first on the various features required the evaluation of the degree of attention, and the accuracy of each feature recognition is as follows. The system performance under different lighting conditions is shown in Table 4.

The test results show that under different lighting conditions, there is almost no difference in the statistical data of natural light conditions and strong light conditions. However, the system’s performance drops significantly under low light conditions, because the system is not sensitive to human faces and eyes under low light conditions, so it is difficult to capture the human face and human eye images in some video frames. The system performance at different distances is shown in Table 5.

Obviously, the performance of the system reaches its best when the experimenter is 30 cm and 50 cm away from the camera, and the performance drops slightly at 70 cm, but most of the eye movement information can still be recognized. When the distance reaches 90 cm and 110 cm, the system performance is significant. The reason is that, when the experimenter is far away from the camera, the angle change required for the gaze to move the same distance on the screen is small, and the resolution of the eye image captured by the camera is low when the distance is far away, so it is difficult to determine the gaze point and gaze direction. However, considering that the distance between learners and cameras is seldom more than 30–50 cm in the online teaching situation, we think that this error will not cause much impact.

### 5.4. Classroom Simulation

Next, we conducted a classroom simulation to test the various indicators of our attention evaluation. We divided 96 postgraduate students into three groups on average and stipulated that their heads should be kept at a position of 50 cm from the screen to study the same course for 20 min. None of the 96 postgraduate students had been contacted about the learning content beforehand. Each participant needs to manually input two data points at the beginning of the experiment, which are the length and width of the screen.

We specified that, during the study period, the first group of students would consistently maintain a high level of concentration on the video content. The second group of students would only focus during the first half of the time and intentionally distract themselves during the second half, while the third group of students would maintain a constant state of distraction. Meanwhile, only the second group of students were asked to wear electrode caps at the beginning of the simulation. We set the attention level of the first group of students to 3, the attention level of the second group to 2, and the attention level of the third group to 1.

**Figure 12:**
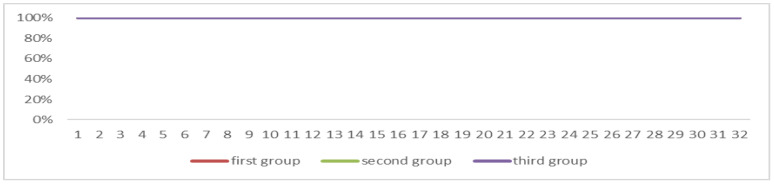
The proportion of the normal frame rate of the face. The POOF values of the first group of students (*n* = 32) are all 100% (the three lines are coincident).

We used seven data items, namely POOF, POHCHP, POMCHP, POEFT, POSEFT, NOS, and NORSP, and used the Spearman correlation coefficient to analyze the relationship between the six data and the degree of concentration. The Spearman correlation coefficient formula is as (7).

(7) $$\rho =\frac{\sum_{i}\left(x_{i}-\bar{x}\right)\left(y_{i}-\bar{y}\right)}{\sqrt{\sum_{i}{\left(x_{i}-\bar{x}\right)}^{2}{\left(y_{i}-\bar{y}\right)}^{2}}}$$

where $x_{i}$ represents the i-th element in the variable *x*, $\bar{x}$ represents the mean value of the variable *x*, and the variable y is the same as *x*. There is no significant difference in POOF among the three groups of students. Due to the setting of the experiment content, the data of the three groups are all 100%, see Figure 12.

**Figure 13:**
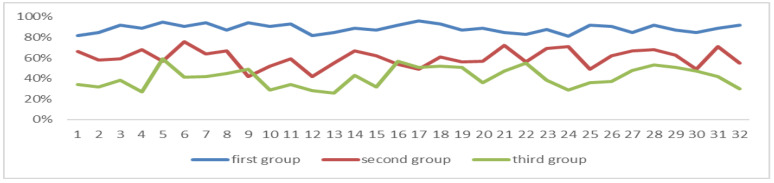
The proportion of frames with an abnormal head pose. The POHCHP values of the first group of students (*n* = 32) are all more than 50%.

The correlation coefficient between POHCHP and the degree of attention among the three groups is 0.9368, and there is a significant relationship between the two (*p* = 0.01), and the first group of data is more than 50%, as can be seen in Figure 13.

**Figure 14:**
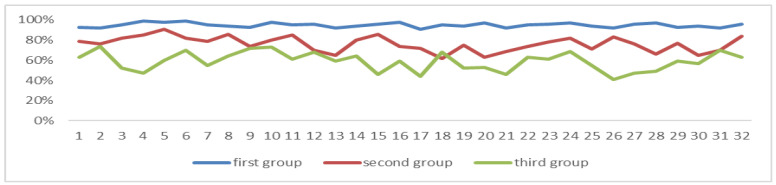
The proportion of frames with an abnormal head pose. The POMCHP values of the first group of students (*n* = 32) are all more than 90%.

The correlation coefficient between POMCHP and the degree of attention among the three groups is 0.8952, and there is a significant relationship between the two (*p* = 0.01), and the first group of data is more than 90%, as can be seen in Figure 14.

**Figure 15:**
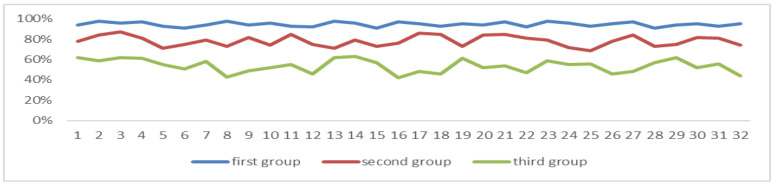
Proportion of the effective fixation time. The POEFT values of the first group of students (*n* = 32) are all greater than 90%.

Among the three groups of students, the correlation coefficient between POEFT and the degree of attention is 0.9489, there is a significant relationship between the two (*n* = 0.01), and the data of the first group are all greater than 90%, as can be seen in Figure 15.

**Figure 16:**
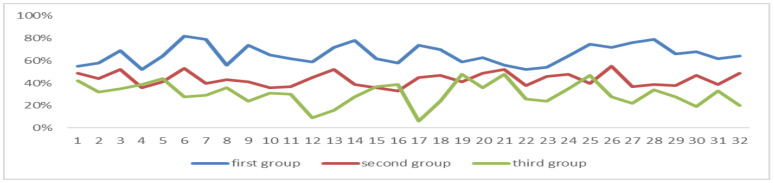
Proportion of serious and effective fixation times. The POSEFT values of the first group of students (*n* = 32) are all greater than 50%.

Among the three groups of students, the correlation coefficient between POSEFT and the degree of attention is 0.8646, and there is a significant relationship between the two (*p* = 0.01), and the data in the first group are all greater than 50%, as can be seen in Figure 16.

**Figure 17:**
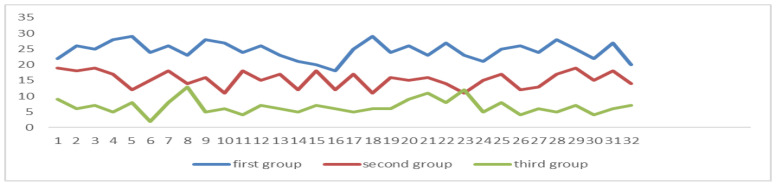
Variance in the number of saccades. The NOS values of the first group of students (*n* = 32) are all less than 10.

The correlation coefficient between the average of NOS and the degree of attention in the three groups was −0.017, and there was no significant relationship between the two (*p* = 0.01). The correlation coefficient between the variance of NOS and the degree of attention is −0.7846 (*p* = 0.01), there is a significant relationship between the two, and the variance of the first group is all less than 10, as can be seen in Figure 17. The average formula is as it is in (8), and the variance formula is as it is in (9).

(8) $$\bar{x}=\frac{\sum_{i}^{n}x_{i}}{n}$$

(9) $$s^{2}=\frac{\sum_{i}^{n}{\left(x_{i}-x\right)}^{2}}{n}$$

$x_{i}$ represents the i-th element in variable *x* and *n* is the number of the sample.

**Figure 18:**
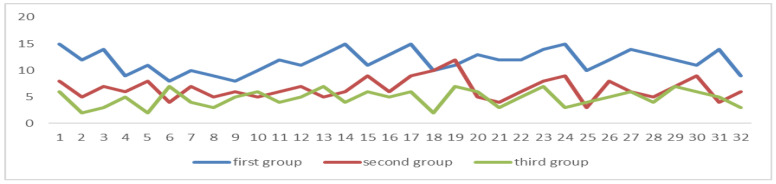
Number of reading smooth pursuit. The NORSP values of the first group of students (*n* = 32) are all greater than 5.

Among the three groups of students, the correlation coefficient between the NORSP and the degree of attention is 0.7687, there is a significant relationship between the two (*p* = 0.01), and the average of NORSP per ten minutes in the first group is greater than 5, as can be seen in Figure 18.

**Figure 19:**
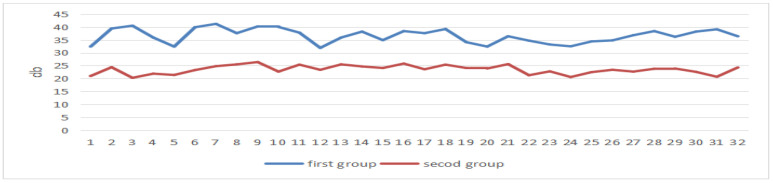
EEG power values of the beta band in two periods.

After the experiment, we processed the EEG data of the students, and the power value of the first half of the EEG beta band of all students was greater than that of the second half, as can be seen in Figure 19.

**Figure 20:**
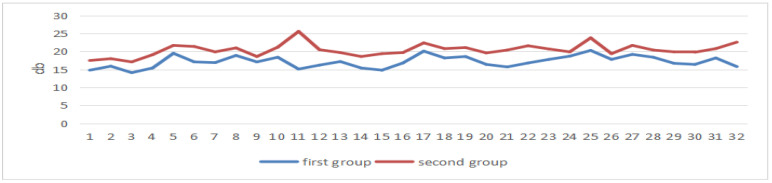
EEG power values of the theta band in two periods.

These two sets of data are the power values of the students’ brain electrical theta band and the values of the first group, which are all lower than those of the second group, see Figure 20.

In the state of concentrated attention, the beta band power increases and the theta band power decreases [54]. From the EEG data, it can be seen that all the students’ attention was more concentrated in the first half of the period than in the second half.

### 5.5. Conclusions

The results of this test show that the statistics of POOF, POHCHP, POMCHP, POEFT, POSEFT, NOS, and NORSP meet the assumptions we made. The second and third groups of students can only meet part of the indicators, and the data of the second group are closer to the threshold we set than the third group. In addition, under the EEG detection, all the students in the second group were relatively concentrated in the first half of the period, and their attention was relatively scattered in the second half. From this, we can conclude that our indicators can evaluate the attention of the audience, and the higher the concentration, the better the performance in various data.

## 6. Summary

In this article, we introduce the important role of eye contact in speech and illustrate the disadvantages of the lack of eye contact in online speech mode. Therefore, we propose an evaluating attention system based on gaze tracking. The gaze tracking part of the system combines face recognition, head pose detection, convolutional neural network, and other technologies to realize the calibration of the user’s gaze characteristics without supporting external equipment. Compared with the traditional eye-tracking method, this method is low in cost, does not require calibration, and allows heads to rotate freely, so it is more suitable for a wide range of applications such as online teaching and virtual meetings.

We have added a new method to analyze the user’s degree of attention in the system. This method is different from the traditional evaluation method that only evaluates the attention based on facial expressions, behaviors, and other characteristics; instead, the degree of attention is analyzed by combining the six indicators of POOF, POHCHP, POMCHP, POEFT, POSEFT, NOS, and NORSP. This method overcomes the inability of speakers to make eye contact with audiences in online speech mode. We validated our approach with classroom simulations, showing that the more focused group met all of our evaluation indicators, while the less focused control group did not. The second group of students was tested with EEG, which verified the accuracy of the system evaluation and also demonstrated that the students could control their own state. However, due to the small sample size, there may be accidental problems in our experimental data. It is necessary to further verify the experimental results through repeated experiments and long-term research. But we hope that this research can be used as a primer to conduct emotion and state analysis by eye movement data, which will play a huge role in related projects. For example, in the field of human–computer interaction and interface design, this system can be utilized to capture user eye movements. Subsequently, when designing applications, websites, or virtual reality environments, the optimization of interface elements can be carried out to enhance user experience. In the field of driving safety and traffic engineering, implementing this system in a vehicle’s internal camera can monitor the driver’s eye movements, allowing for the assessment of their attention level and fatigue. This is crucial for improving driving safety and preventing traffic accidents. In the field of medical engineering, the system can be employed to evaluate a patient’s attention levels, particularly in operating rooms or clinical settings. By monitoring the eye movements of healthcare professionals or patients, surgical safety and efficiency can be improved. In the field of education, the system can be used to analyze students’ attention levels during the learning process. Educational technology companies can design personalized learning experiences based on students’ eye movement characteristics, thereby enhancing teaching effectiveness. In the field of industrial production, the system can monitor workers’ eye movements to help optimize production processes and improve the overall work efficiency. Additionally, assessing workers’ attention levels can reduce operational errors and enhance workplace safety. Further research may develop different criteria for every people through the individualized analysis of audiences. Since individuals have differences in habits, learning abilities, etc., the evaluation of different individuals can adjust the threshold in our evaluation indicators, so that each individual can be more accurately evaluated. In addition to that, we are planning to add a feature in our program that can read the device model, allowing our system to automatically retrieve the screen size of the user’s device. We can also further expand our index system by combining features such as pupils and irises to make our research results more convincing.

At the same time, our proposed attention evaluation method still has some limitations. The specific application needs to be arranged on the subject’s client, and where the collected data are stored needs further specific consideration. Eye movement data processing on the device has a certain timeliness gap compared to the offline teaching eye contact. In addition, this paper is a preliminary study of this topic, and large-scale applications will certainly reveal more problems. More targeted solutions will hopefully bring certain contributions to society in the future.

## Figures and Tables

**Table 1:** Comparison chart of existing gaze-tracking algorithms.

Method	Advantage	Disadvantage
**Gaze tracking based** **on geometry**	The system structure is simple and does not require complicated calibration	The head needs to maintain a fixed posture and cannot move widely
**Gaze tracking based** **on 3D eye model**	Allowing head movement	Calibration work is required
**Gaze tracking based** **on eye appearance features**	The accuracy requirements for equipment are low	Difficult to overcome the influence of light

**Table 2:** The confusion matrix of GazeNet.

		Predicted Value	
		1	0
**True value**	1	10,218	1237
	0	1947	7598

**Table 3:** The confusion matrix of GazeNet.

		Predicted Value	
		1	0
**True value**	1	8129	1213
	0	1887	8794

**Table 4:** System performance under different lighting conditions.

	POOF	POHCHP	POMCHP	POEFT	POSEFT	NOS	NORSP
**Low light**	100%	100%	100%	90%	87%	92%	72%
**Natural light**	100%	100%	100%	95%	93%	98%	87%
**Strong light**	100%	100%	100%	96%	92%	98%	86%

**Table 5:** System performance test at different distances.

	POOF	POHCHP	POMCHP	POEFT	POSEFT	NOS	NORSP
**30 cm**	100%	100%	100%	99%	95%	98%	85%
**50 cm**	100%	100%	100%	100%	95%	97%	87%
**70 cm**	100%	100%	100%	97%	96%	89%	86%
**90 cm**	100%	100%	100%	89%	84%	51%	42%
**110 cm**	100%	100%	100%	75%	69%	14%	10%

## Data Availability

All data generated or analyzed during this study are included in this published article.
